# Evaluating Personalized Add-On Ayurveda Therapy in Oxygen-Dependent Diabetic COVID-19 Patients: A 60-Day Study of Symptoms, Inflammation, and Radiological Changes

**DOI:** 10.7759/cureus.68392

**Published:** 2024-09-01

**Authors:** Somit Kumar, Karthikeyan Ramaraju, Mitravinda S Kakarla, Sujith S Eranezhath, Chaithanya Chenthamarakshan, Murali Alagesan, Balagopal Satheesan, Indulal Unniappan, Holly Wilhalme, Valdis Pīrāgs, Daniel E Furst

**Affiliations:** 1 Clinical Research, AVP Research Foundation, Coimbatore, IND; 2 Research and Development, The Arya Vaidya Pharmacy, Coimbatore, IND; 3 Respiratory Medicine, PSG Institute of Medical Sciences and Research, Coimbatore, IND; 4 Statistics, AVP Research Foundation, Coimbatore, IND; 5 Basic and Translational Research, AVP Research Foundation, Coimbatore, IND; 6 Ayurveda and Integrative Medicine, AVP Research Foundation, Coimbatore, IND; 7 General Medicine, PSG Institute of Medical Sciences and Research, Coimbatore, IND; 8 Ayurveda and Integrative Medicine, Saranya Ayurveda Hospital, Coimbatore, IND; 9 Statistics, University of California Los Angeles, Los Angeles, USA; 10 Medicine, University of Latvia, Riga, LVA; 11 Rheumatology, University of California Los Angeles, Los Angeles, USA; 12 Rheumatology, University of Washington, Seattle, USA; 13 Rheumatology, University of Florence, Florence, ITA

**Keywords:** innate immunity, sannipata jwara, c-reactive protein, cytokine interleukin-6, neutrophils-lymphocyte ratio

## Abstract

Background

Effective management of both acute and post-acute sequelae of SARS-CoV-2 is essential, particularly for type 2 diabetes mellitus (T2DM) patients, who are at increased risk of severe pro-inflammatory responses and complications. Persistent symptoms and residual lung and cardiovascular damage in post-coronavirus disease (COVID-19) individuals highlight the need for comprehensive long-term treatment strategies. Conventional treatments, including Remdesivir and glucocorticoids, have limitations, suggesting that further investigation into Ayurvedic therapies could be beneficial, though controlled trials are currently limited.

Objectives

Evaluate the effectiveness and safety of Ayurveda with the standard of care (SOC) versus SOC in improving symptoms, moderating immune responses (interleukin-6 (IL-6), C-reactive protein (CRP), neutrophil-lymphocyte ratio (NLR), and radiological outcomes in oxygen-dependent, high-risk, non-vaccinated type 2 diabetes COVID-19 patients over 60 days, and thus addressing their heightened vulnerability to severe infections.

Methods

A controlled trial with 50 diabetic COVID-19 patients, aged 18-80, with an NLR of >= 4, primarily on Remdesivir, was assigned to Group 1 (Add-on Ayurveda+SOC, n=30) or Group 2 (SOC, n=20) based on their voluntary choice with follow-up on days 14, 28, and 60. Parametric outcomes in group analysis were assessed with robust regression and non-parametric outcomes with Cochran-Mantel-Haenszel, log-rank test, and chi-square tests at 95% confidence interval (CI).

Results

Group 1 exhibited statistically significant improvements in fever, cough, diarrhea, as well as NLR, IL-6, and CRP by 14 days, and in anosmia, loss of taste, shortness of breath, general weakness, and headache by 60 days. Though the sample size is small, notable improvements can be seen in troponin levels in Group 1 at 28 and 60 days. High-resolution computer tomography COVID-19 reporting and data system (HRCT CO-RADS) scores improved more slowly in Group 2 than in Group 1. Survival rates were 96.4% for Group 1 and 90% for Group 2. Numbers were too small for reliable comparisons at 60 days.

Conclusion

The add-on Ayurveda group showed a better symptomatic response, and faster normalization in inflammatory markers, including IL-6 and NLR by 14 days, and cardiac markers by 28 days. Minimal clinical and no laboratory adverse events were observed. This study supports the need for a randomized, double-blind trial.

## Introduction

The implementation of robust and effective multi-disciplinary strategies to address the acute and post-acute sequelae of SARS-CoV-2 infection is needed, as repeated waves are expected over time. Type 2 diabetes mellitus (T2DM) individuals diagnosed with COVID-19 are at an increased risk of experiencing an exacerbated pro-inflammatory response secondary to dysregulated innate immunity and compromised cell-mediated immune response [1]. Such cases often necessitate mechanical ventilation and ICU admission, thereby contributing to higher mortality rates [2]. The situation is complicated by the presence of comorbidities such as hypertension, chronic kidney disease, and coronary artery disease, which are commonly associated with type 2 diabetes [3-5]. Of interest, these patients have elevated neutrophil-to-lymphocyte ratios, indicating inflammation, and importantly, elevated high-sensitivity C-reactive protein and interleukin-6 levels in the initial phases of the infection [6].

Post-acute sequelae following SARS-CoV-2 infection manifest as persistent symptoms and organ damage in individuals who have recovered from the acute phase of COVID-19. These enduring symptoms include but are not limited to, shortness of breath, cognitive dysfunction, anxiety, depression, muscle aches, fever, loss of smell, loss of taste, and specific damage to the heart and kidneys, with a notable focus on lung fibrosis marked by pathological scarring of pulmonary tissues [7]. The HRCT CORADS score can help identify residual lung damage and provide insights into the severity and progression of the disease [8,9]. Multiple studies have demonstrated that individuals without preexisting cardiovascular conditions who contract COVID-19 can exhibit cardiac complications or myocardial injury [10]. Therefore, it is of scientific interest to investigate how various therapies influence long-term cardiac complications in such cases.

Allopathic COVID-19 treatments include Remdesivir, which reduces hospital stays but does not reliably alleviate symptoms and can affect liver and renal function [11-14]. Glucocorticoids lower 28-day mortality for patients on ventilation or oxygen but prolong viral shedding [15-17]. Glucocorticoids do not decrease mortality in those not requiring ventilation or oxygen, and high doses could actually increase mortality compared to standard care [16,17]. Paxlovid reduces hospitalization and mortality, but its effectiveness against variants and severe cases is uncertain [18]. Tocilizumab, an IL-6 receptor inhibitor, reduces mortality and complications for some patients, but not when combined with steroids [19,20]. Convalescent plasma and IVIG have limited efficacy and may pose risks [21].

Ayurveda, a traditional medicine system, adopts a distinct epistemological approach supported by both controlled and uncontrolled evidence demonstrating the effectiveness of using adjuvant Ayurvedic herbs in treating SARS-CoV-2 infections [22-25]. Case reports and case series on integrating Ayurveda in treating diabetic COVID-19 patients exist, but controlled trials are very limited [25-33].

The objective of this study is to address the acute and post-acute sequelae of add-on Ayurveda in non-vaccinated, hospitalized, oxygen-dependent pre-existing Type II diabetes mellitus patients in moderate to severe COVID-19. This intervention is centered around addressing the early accumulation of cytokines, particularly targeting interleukin-6 (IL-6) and C-reactive protein (CRP) as indicators of dysregulated innate immune response. Additionally, it focuses on assessing the neutrophils-lymphocytes ratio (NLR), an indicator of both innate and cell-mediated immune responses. Further, the study explores the effectiveness of the intervention in moderating laboratory markers, lung fibrosis (high-resolution computed tomography [HRCT]), cardiac damage (HS-troponin), renal (renal function tests), and hepatic (liver function tests) through a longer follow-up period.

## Materials and methods

Study design

A 60-day, open-label, two-arm, controlled study involving RT-PCR-positive COVID-19 type II diabetes mellitus patients receiving oxygen support but not requiring ICU admission at the time of induction (i.e., moderate severity) was initiated. The study compared Group 1 (Standard of Care (SOC) plus personalized add-on Ayurveda intervention) against Group 2 (SOC only) for up to 60 days. SOC was defined as any treatment that adhered to the "ICMR Guideline on Clinical Management in COVID Treatment Facilities" (used in both groups 1 and 2) [34].

Patients

At the special COVID wards of the PSG Institute of Medical Sciences and Research Hospital in Coimbatore, India, 9,887 hospitalized individuals suspected of COVID-19 underwent screening. Among them, 236 unvaccinated diabetic individuals who tested positive for COVID-19 via RT-PCR were invited to participate in the study. The lack of vaccination among these patients was due to either personal choice or challenges in accessing vaccination drives before contracting the virus. The study initially aimed to enroll 100 individuals who met the inclusion and exclusion criteria, commencing in January 2021 on an exploratory basis. However, only 50 unvaccinated participants consented to participate: 30 in Group 1 (received add-on Ayurveda + standard of care) and 20 in Group 2 (received only standard of care). The participants were allocated based on their own voluntary choice into either of the groups. Despite our extensive efforts to achieve the target sample size, recruitment was impeded by the progression of the vaccination drive, decreased severity of infections, and stringent inclusion criteria by January 2022. Including vaccinated subjects in the mid-study could have introduced variability, created subgroups, and obstructed a clear understanding of the primary innate immune response. Thus, this is a controlled but not a randomized study. Group 1 received personalized Ayurvedic treatments, while Group 2 received as per standard of care without Ayurvedic drugs. Both groups were prescribed medications based on symptoms and clinical assessments during follow-up. The rationality behind Ayurvedic and allopathic medications used is furnished in the Appendix (Tables 15-19). The study was registered in the Clinical Trial Registry of India (CTRI Reg No: CTRI/2020/12/029985) and approved by the PSG Institute's Institutional Human Ethics Committee (No: PSG/IHEC/2020/Appr/FB/034). The study period was from December 28, 2020, to January 21, 2022. Ultimately, only 26 out of the 50 unvaccinated participants completed the study due to follow-up challenges, such as transportation issues for patients from other cities, psychological barriers like fear of being identified as a COVID patient or reinfection, a lack of awareness about the importance of follow-up, personal health concerns, and family commitments.

Specific aims

Primary Outcome Measures

To examine the symptomatic response of the patients (fever, anosmia, loss of sense of taste, cough, dyspnea, sore throat, rhinorrhoea, general weakness, headache, irritability, confusion, nausea, and diarrhea) treated with personalized add-on integrative therapy. compared to SOC alone on Baseline, day 14, day 28, and day 60.

To examine the biomarker response (serum IL-6, CRP, NLR) of the patients treated with add-on integrative therapy compared to SOC alone on baseline, day 14, day 28, and day 60.

Secondary Outcome Measures

To examine changes in random blood sugar, hemoglobin, WBC, RBC, absolute neutrophils, absolute lymphocytes, absolute monocytes, platelet count, ALT, AST, ALP, albumin, total bilirubin, direct bilirubin, prothrombin time, serum creatinine, urea, LDH, ferritin, D-dimer, troponin, and HRCT of the lungs.

The detailed schedule of events for primary and secondary outcomes was followed. All lab investigations for patients in the study were performed at the same NABL-accredited laboratory, thereby reducing interlaboratory variability.

Inclusion criteria

The study included participants who were confirmed COVID-19 cases requiring oxygen supplementation but did not need ventilator or ICU support and were aged between 18 and 80 years. Eligible individuals were willing to provide consent and had a pre-existing diagnosis of type II diabetes mellitus. Patients had SpO2 levels between 90-94% on room air or less than 90% with prior approval and a neutrophil-to-lymphocyte ratio (NLR) of 4 or higher. Additionally, participants were supposed to be on Remdesivir medication primarily and were non-vaccinated.

Exclusion criteria

The study excluded individuals with clinically diagnosed COPD, active malignancy, chronic liver or renal diseases, or those who had experienced a myocardial infarction (MI) or cerebrovascular accident (CVA) within the past year. Also excluded were those with type I diabetes mellitus, COVID-19-induced hyperglycemia, or those who were on the faculty or staff of the participating institutions.

Treatment

Throughout the 60-day study period, the interventions were closely monitored and adjusted as needed based on clinical requirements. Both study arms followed the Standard 'ICMR Guideline on Clinical Management in COVID-19'. The Ayurveda interventions in the study group involved intensive drug-based treatments along with dietary and lifestyle modifications, following the principles of classical Ayurveda for distinct stages of *Sannipāta jvara* (complex fever). During hospitalization, diet and lifestyle conformed to conventional protocols typically adhered to during inpatient care. Ayurveda-based diet and lifestyle suggestions were recommended post-discharge and continued until the 60th day. The personalized intervention was formulated utilizing a host-centric approach, targeting distinct stages of *Sannipāta jvara* (complicated fever): *Nava jvara* (acute stage of fever), *Pachyamana jvara* (phase between ama stage and post-ama stage of fever), *Jirna jvara* (chronic fever), and some cases of COVID-19 demonstrating features of *Punaravartaka jvara* (repeated fever). The primary emphasis was on addressing the initial *Dhatu-gata jvara lakshana* (fever/inflammation in body elements) with the intention of preventing its progression to *Dhatu-paka* (tissue damage) and mitigating the associated risk of *Ojo Kshayam* (an immunocompromised state). This strategic approach was aimed at reducing the potential for increased mortality. Specifics of the Ayurveda intervention, based on *Agni* (gut and cellular metabolism), *Vikrithi* (disease phenotype), and *samprapti* (pathogenesis) stages, are outlined in Table 1 and Appendix (Tables 15-18). The medications administered in the study group were exclusively comprised of standardized classical Ayurveda formulations derived from the classical textbooks specified in the First Schedule of the Drugs and Cosmetics Act. These formulations were manufactured following the preparation guidelines outlined in the Ayurvedic Formulary of India (AFI). They included *Kashayam* (concentrated water decoction), *Gulikas* (processed Ayurvedic herbo-mineral pills), *Arishtas*, and *Asavas* (fermented herbal juices and concentrated water decoctions), *Ghritam* (clarified butter processed in herbal juices and decoctions), and *Choornas* (pulverized herbal powder). Notably, no patented, plant-extract-based medicines or proprietary medications were employed in the study. The classical, non-patented Ayurveda formulations underwent rigorous quality control assessments to ascertain their safety and standardization.

**Table 1:** Details of the medicines used in each group *Only the front-line medications as given in the ICMR guidelines have been mentioned above. For a detailed medicine chart, refer to Appendix (Tables 15-18)

Medicine List	Number of people receiving the medication	
	Add-on Ayurveda Group (n = 30)	Standard of care Group (n=20)
Ayurvedic formulations		
Indukantham Kashayam	27	-
Bharangyadi Kashayam	27	-
Panchathikthakam Kashayam	22	-
Drakshadi Kashyam	17	-
Guduchyadi Kashayam	9	-
Vilwadi Gulika	2	-
Patolakadurohinyadi Kashayam	1	-
Pravala Bhasmam	23	-
Swasanandam Gulika	24	-
Gorochanadi Gulika	27	-
Suvarnamukatadi Gulika	26	-
Brahmarasayana	11	-
Agasthyarasayanam	24	-
Dhanwantharam Gritham	3	-
Vidaryadi Lehyam	7	-
Baladi Gritham	20	-
Nayopayam Kashayam	15	-
Rasonadi Kashayam	2	-
Allopathic medicines prescribed as per ICMR Guidelines*		
Inj.Remdesivir	30	20
Inj.Clexane	25	19
Inj.Pan	27	19
Inj.Methylprednisolone	19	18
T.Dolo	26	17
T.Zac D	21	17
Syp.Alex	16	11
T.Metformin	20	10
T.Glimepiride	6	1
Inj.Actrapid	5	2
T.Vildagliptin	5	2
T.Glycomet	4	3
Inj.Insulin	4	2
Inj.Mixtard	4	0
T.Janumet	3	0
T.Gluconorm	2	2
T.Gemer	2	1
T.Gliclazide	2	1
T.Teneligliptin	2	1
T.Diamicron	2	0
T.Glycinorm	2	0
T.Voglibose	1	2
Inj.Cantus	1	0
Inj.Lantus	1	0
T.Istamet	1	0
T.Jalra M	1	0
T.Lupisulin	1	0
T.Obimet	1	0
T.Oxra	1	0
T.Pioglitazone	1	0
T.Reclide	1	0
T.Reclimet	1	0
T.Valera	1	0
T.Vilzatom	1	0
T.Vinglyn	1	0
T.Vogs Gm-2	1	0

Statistical analysis

The analysis included available subjects at each follow-up visit, incorporating data from dropouts. The final analysis was performed using SAS 9.4 software, which comprised 30 patients in Group 1 and 20 in Group 2, with an 80% compliance rate (based on active medication days within the study period) and no major protocol deviations. Efficacy between the groups for continuous outcomes was assessed using robust regression, which accounted for outliers and deviations from normality while adjusting for baseline differences. For dichotomous primary symptomatic outcomes, the Cochran-Mantel-Haenszel test was applied, factoring in baseline variations. To address the impact of unequal sample sizes between the groups, weighted analyses were employed. Trend curves, which included all patients at each eligible visit, were used to visualize data changes over time for both the treatment and control groups.

A sensitivity analysis was performed to evaluate the influence of missing data on significant outcomes, utilizing the last observation carried forward (LOCF) method under the missing at random (MAR) assumption. This analysis employed robust regression, ANCOVA, and mixed-effects logistic regression models as appropriate. All statistical tests were conducted with a significance level of 0.05.

## Results

Baseline characteristics

Among the 50 enrolled patients (30 in Gp 1; 20 in Gp 2), 44 completed 14 days (25 in Gp 1; 19 in Gp 2), 30 completed 28 days (19 in Gp 1; 11 in Gp 2), and 26 completed 60 days (17 in Gp 1; 9 in Gp 2). Notably, there were no statistically significant differences in demographic characteristics, baseline biomarkers, or presenting symptoms between the two groups. The mean age (Gp 1: 58.9 ± 9.5 yrs vs. Gp 2: 60.15 ± 9.4 yrs, p = NS), gender distribution (Gp 1: 70% males vs. Gp 2: 60% males, p = NS), random blood sugar levels (Gp 1: 259.64 ± 107 mg/dL vs. Gp 2: 254.83 ± 124.58 mg/dL, p = NS), and duration of diabetes (Gp 1: 9.0 ± 6.3 yrs vs. Gp 2: 10.8 ± 6.09 yrs, p = NS) demonstrated no significant differences. Both the add-on Ayurveda group and the Standard of Care group had a median hospitalization duration of eight days. Predominant symptoms, namely cough (Gp 1: 46.7%, vs. Gp 2: 40%, p = NS), fever (Gp 1: 46.7%, vs. Gp 2: 45%, p = NS), and dyspnea (Gp 1: 26.7%, vs. Gp 2: 25%, p = NS), were reported as first symptoms of COVID-19 by a majority of participants in both groups. The time to onset of these symptoms was 5-7 days (median). Baseline assessments, including NLR (Gp 1: 7.88 ± 3.81 vs. Gp 2: 7.19 ± 2.8, p = NS), and HRCT CO-RADS scores (Gp 1: 12.66 ± 4.38 vs. Gp 2: 12.21 ± 4.45, p = NS), were not different between the groups. Furthermore, the proportions of participants receiving Remdesivir and Methylprednisolone were similar (Gp 1: 93.3% vs. Gp 2: 95%, p = NS). Detailed information on baseline demographic characteristics is provided in Table 2.

**Table 2:** Clinical demography Gp 1: Add on Ayurveda Group, Gp 2: Standard of Care Group A: Values assessed by parametric tests (Independent Samples t-test). B: Values assessed by non-parametric tests (Chi-Square test, Median test, Proportions (Z) test, and Wilcoxon’s Rank-Sum test)

	Gp 1	Gp 2	p-value
Total subjects (at baseline)	30	20	
Male	21 (70%)	12 (60%)	0.63^b^
Female	9 (30%)	8 (40%)	
Age	58.86 (±9.52)	60.15 (±9.38)	0.64^a^
Male	59.5 (±8.99)	57.5 (±9.99)	
Female	57.3 (±11.1)	64.12 (±7.22)	
No of subjects with history of co-morbidities			
Diabetes	30 (100%)	20 (100%)	
Duration (in years)	9 (±6.3)	10.8 (±6.09)	0.19^ b^
Hypertension	13 (43.3%)	10 (50%)	
Chronic cardiac disease	3 (10%)	3 (15%)	
Smoking status	4 (13.3%)	2 (10%)	
Current	1	1	
Past	3	1	
Hypotension	1 (3.3%)	0 (0%)	
Hypothyroidism	1 (3.3%)	0 (0%)	
Asthma	0 (0%)	1 (5%)	
Baseline oxygen saturation	94 (89 - 96.75)	94.5 (92.25 - 96.75)	0.43^b^
Baseline CT scores	12.66 (±4.38)	12.21 (±4.45)	0.72^b^
Baseline neutrophils to lymphocytes ratio	7.88 (±3.81)	7.19 (±2.80)	0.75^ b^
Baseline random blood sugar	259.64 (±107)	254.83 (±124.58)	0.66^ b^
No of subjects at each follow up visit			
14 days follow up	25	19	
28 days follow up	19	11	
60 days follow up	17	9	
Median number of days of hospitalization	8 (6 - 12)	8 (6 - 12)	0.99^ b^
Number of subjects on Remdesivir and Methylprednisolone	28 (93.3%)	19 (95%)	0.99^ b^
Onset of first symptom – median duration			
Cough	14 (46.7%) - 7 days	8 (40%) - 7 days	0.84^ b^
Fever	14 (46.7%) - 7 days	9 (45%) - 5 days	0.09^ b^
Dyspnea	8 (26.7%) - 5.5 days	5 (25%) - 6 days	0.80^ b^
Fatigue	5 (16.7%) - 7 days	6 (30%) - 6 days	0.41^ b^
Tastelessness	3 (10%) - 5 days	1 (5%) - 3 days	
Anosmia	2 (6.7%) - 4 days	3 (15%) - 2.5 days	
Nausea	2 (6.7%) - 9.5 days	3 (15%) - 4.5 days	
Diarrhea	1 (3.3%) - 7 days	6 (30%) - 3.5 days	
Headache	1 (3.3%) - 7 days	2 (10%) - 7 days	
Running Nose	1 (3.3%) - 5 days	0 (0%)	

Assessment of markers on day 14

**Figure 1:**
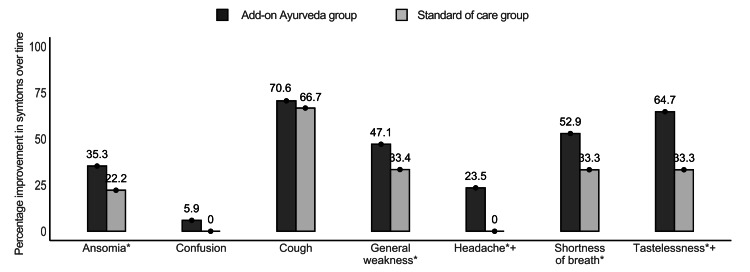
Alleviation in symptomatic parameters over 14 and 60 days (percentage decrease) * Depicts statistical significance between the groups at 60 days assessed by Cochran-Mantel-Haenszel test after adjusting for baseline values. p-value=0.00-0.04 for all + Depicts statistical significance between the groups at 14 days assessed by Chi-square/Fisher’s test. p-value=0.00-0.04 for all

**Figure 2:**
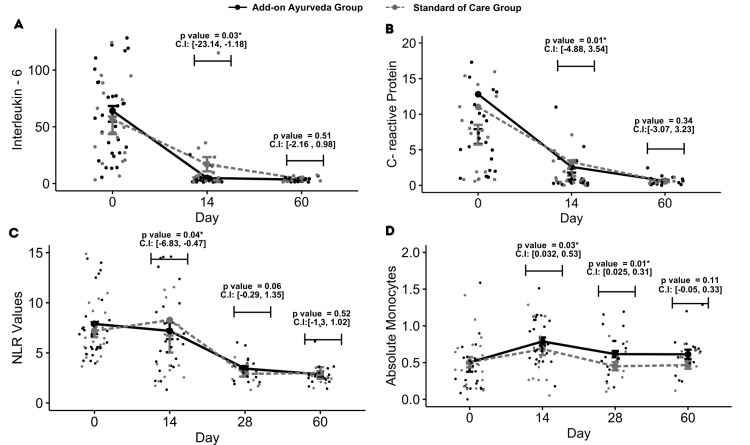
Changes in primary outcomes A, B, C, D depicts mean value of patients over time in add-on ayurveda group and standard of care for IL-6, CRP, neutrophils to lymphocytes ratio, and absolute monocytes. *p-value computed using robust regression controlling for baseline differences between group on the particular visit.

The analysis revealed significant differences between groups across several symptomatic outcomes, namely loss of taste (p = 0.01), cough (p = 0.01), shortness of breath (p = 0.01), and fever (p = 0.04), which exhibited marked improvements within the add-on Ayurveda group. Diarrhea (p = 0.02) exhibited improvements in the standard of care. Other symptoms like anosmia, general weakness, sore throat, irritability, confusion, and nausea displayed consistent patterns across both groups (Tables 3, 4, Figure 1). Robust regression analysis demonstrated a notable decrease in primary biomarkers, namely neutrophil-to-lymphocyte ratio (NLR) (p = 0.04), interleukin-6 (IL-6) levels (p = 0.03), and C-reactive protein (CRP) levels (p = 0.01) within the add-on Ayurveda group (Table 5, Figure 2). Additionally, significant improvements in absolute monocytes (p = 0.03) were observed in the add-on Ayurveda group (Figure 2). Similar results were observed by sensitivity analysis, which accounted for the imputed data.

**Table 3:** Primary outcomes: Alleviation in sub-acute symptomatic parameters Gp 1: Add on Ayurveda Group, Gp 2: Standard of Care Group *Values at 95% confidence interval where the level of significance, α= 0.05. A: Values assessed by Cochran-Mantel-Haenszel test after adjusting for baseline difference. B: Values assessed by Chi-square/Fisher’s test over 14 days.

Parameter	Baseline		Day 14			Day 28			Day 60		
	Gp 1 (N=17)	Gp 2 (N = 9)	Gp 1 (N=17)	Gp 2 (N = 9)	p value over 14 days^b^	Gp 1 (N=17)	Gp 2 (N = 9)	p value over 28 days^a^	Gp 1 (N=17)	Gp 2 (N = 9)	p value over 60 days^a^
Anosmia	7 (41.2%)	2 (22.2%)	3 (17.6%)	1 (11.1%)	0.06	2 (11.8%)	0 (0%)	0.07	1 (5.9%)	0 (0%)	0.04*
Loss of taste	12 (70.6%)	3 (33.3%)	3 (17.6%)	1 (11.1%)	0.01*	2 (11.8%)	0 (0%)	0.03*	1 (5.9%)	0 (0%)	0.02*
Cough	15 (88.2%)	7 (77.8%)	7 (41.2%)	3 (33.3%)	0.01*	7 (41.2%)	1 (11.1%)	0.59	3 (17.6%)	1 (11.1%)	0.28
Shortness of breath	10 (58.8%)	4 (44.4%)	4 (23.5%)	1 (11.1%)	0.01*	3 (17.6%)	1 (11.1%)	0.03*	1 (5.9%)	1 (11.1%)	0.03*
General Weakness	13 (76.5%)	6 (66.7%)	9 (52.9%)	4 (44.4%)	0.622	6 (35.3%)	2 (22.2%)	0.06	5 (29.4%)	3 (33.3%)	0.02*
Headache	5 (29.4%)	1 (11.1%)	1 (5.9%)	0 (0%)	0.02*	5 (29.4%)	0 (0%)	0.04*	1 (5.9%)	1 (11.1%)	0.03*
Confusion	2 (11.8%)	0 (0%)	0 (0%)	0 (0%)	0.33	1 (5.9%)	0 (0%)	-	1 (5.9%)	0 (0%)	-

**Table 4:** Primary outcomes: Alleviation in acute symptomatic parameters over 14 days Gp 1: Add on Ayurveda Group, Gp 2: Standard of Care Group *Values at 95% confidence interval where the level of significance, α= 0.05. A: Values assessed by Chi-square/Fisher’s test over 14 days.

Parameter	Baseline		Day 14		
	Gp 1 (N=24)	Gp 2 (N = 19)	Gp 1 (N=24)	Gp 2 (N = 19)	p-value over 14 days^ a^
Fever	5 (20.83%)	2 (10.53%)	0 (0%)	0 (0%)	0.04*
Sore throat	2 (8.33%)	4 (21.06%)	5 (20.83%)	0 (0%)	0.06
Running Nose	2 (8.33%)	0 (0%)	0 (0%)	1 (5.26%)	
Irritability	4 (16.67%)	4 (21.06%)	1 (4.16%)	1 (5.26%)	0.99
Nausea	5 (20.83%)	6 (31.58%)	1 (4.16%)	0 (0%)	0.99
Diarrhea	2 (8.33%)	8 (42.08%)	1 (4.16%)	0 (0%)	0.02*

**Table 5:** Primary outcomes: Interleukin – 6, C-reactive protein, neutrophils to lymphocytes ratio Gp 1: Add on Ayurveda Group, Gp 2: Standard of Care Group * Values at a 95% confidence interval where the level of significance, α= 0.05 The method of Robust regression controlled for baseline differences with treatment group as covariate has been used to testing differences between the treatment groups. ** Parameter not assessed on day 28 as per the protocol; NA: Not applicable

Parameter	Days of therapy	Gp 1			Gp 2			p-value	Estimate
		N	Mean	SD	N	Mean	SD		
Interleukin -6	0	30	64.05	39.38	20	56.75	40.95		
	14	25	4.85	5.23	18	17.01	26.61	0.03*	-1.79
	28	NA**	NA**	NA**	NA**	NA**	NA**	NA**	NA**
	60	16	3.43	1.52	9	4.02	2.28	0.51	-0.53
C-Reactive Protein	0	26	12.80	21.88	18	11.01	14.26		
	14	25	2.60	6.90	17	3.27	6.18	0.01*	-0.5
	28	NA**	NA**	NA**	NA**	NA**	NA**	NA**	NA**
	60	16	0.62	0.56	9	0.54	0.28	0.34	-0.12
Neutrophils-to-Lymphocytes Ratio	0	30	7.88	3.81	20	7.19	2.80		
	14	25	7.00	4.13	19	10.65	6.30	0.04*	-3.09
	28	19	3.43	1.12	11	2.90	0.92	0.06	0.47
	60	16	2.86	1.07	9	3.00	1.74	0.52	0.20

Assessment of markers on day 28

On this day, significant differences from baseline were noted in certain symptoms: loss of taste (p = 0.03), headache (p = 0.04), and shortness of breath (p = 0.03), favoring the add-on Ayurveda group. However, symptoms like anosmia, general weakness, and cough showed similar trends in both groups (Table 3, Figure 1). The neutrophil-to-lymphocyte ratio (NLR) didn't differ significantly between groups, indicating improvement in both. Troponin levels were notably higher in the standard of care (SOC) group (p = 0.01). Additionally, absolute monocytes (p = 0.01) and total monocytes (p = 0.01) significantly improved within the add-on Ayurveda group (Appendix [Table 10]).

Assessment of markers on day 60

**Figure 3:**
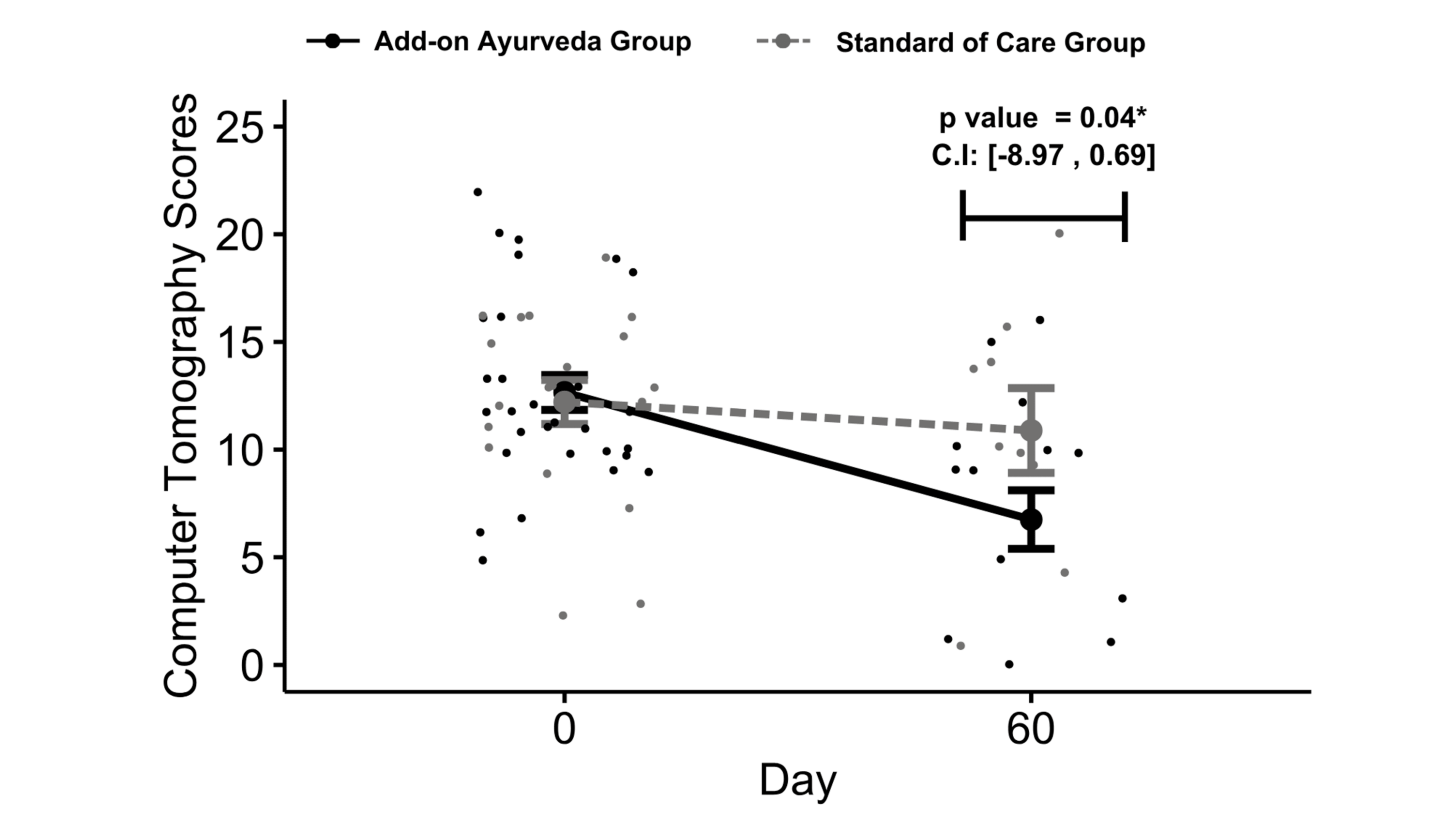
Changes in CT scores *p-value computed using robust regression controlling for baseline differences

The add-on Ayurveda group exhibited statistically significant improvements in symptoms from baseline, including anosmia (p=0.04), loss of taste (p=0.02), shortness of breath (p=0.03), general weakness (p=0.02), and headache (p=0.03). Cough demonstrated a similar response between the groups (Table 3, Figure 1) Troponin levels and platelet counts were notably higher in the SOC group (p = 0.01 each) (Appendix [Table 10]). Furthermore, HRCT CO-RADS scores displayed a significant decrease in the add-on group compared to SOC (Delta Gp 1 = 47%, Delta in Gp 2 = 10.8%, p = 0.04) (Figure 3).

Impact on hematological and biochemical markers

Random blood sugar levels displayed a random pattern with no statistically significant variances observed between the groups (p = NS) over 60 days (Appendix [Table 11]). No consistent or clinically meaningful differences were observed in hemoglobin, WBC, AST, ALT, albumin, total bilirubin, alkaline phosphatase, creatinine, and ferritin levels, or in the vital signs (Appendix [Tables 11-13]). Survival rates did not differ significantly between Gp 1 (96.37%) and Gp 2 (90%) (p = NS) (Appendix [Table 14]). The sensitivity analysis, employing multiple imputations, is in accordance with the results obtained from the primary analyses.

Adverse effects

In Gp 1 (add-on Ayurveda), four out of 30 enrolled patients experienced nine adverse events (AEs). These included two gastrointestinal (blister formation on lips, constipation), one cardiovascular (pedal edema), one ENT (burning sensation in eyes), one skin (itching), one urinary tract (frequent urination), two respiratory (breathing difficulty, traces of blood in sputum), and one general systemic symptom (sweating). Additionally, there were two serious adverse events (SAEs), with one patient dying of disease and another requiring transfer to intensive care due to low oxygen saturation but eventually recovering. In Gp 2 (Standard of Care), one out of 20 patients had a moderate drug-related cardiovascular AE (pedal edema), and there were two SAEs resulting in patient deaths due to the progression of their underlying disease.

The complete adverse event details based on the body system and treatment group can be found in the Appendix (Table 9).

Results of sensitivity analysis

The sensitivity analysis indicated that the outcomes, IL-6, CRP, and NLR, remained consistent with the main analysis over the 60-day period. Secondary outcomes, including H-S troponin and HRCT scores, also demonstrated no significant differences when compared to the main analysis. However, slight variations were observed in symptomatic outcomes.

The analysis showed statistically significant differences between groups at day 14 for fever and anosmia, with improvements noted in the add-on Ayurveda group. By day 28, anosmia showed better improvement in the add-on Ayurveda group. By day 60, both anosmia and dyspnea demonstrated enhanced improvement in the add-on Ayurveda group (p<0.04 for all). Other symptoms showed similar improvements between both groups (Appendix [Tables 6-8]). The sensitivity analysis, despite showing lower statistical significance for symptomatic outcomes, still indicated trends and patterns consistent with the main analysis.

## Discussion

In this controlled study, the addition of Ayurvedic therapy to standard treatment (remdesivir/methylprednisolone) for moderately severe COVID-19 patients with type 2 diabetes was found to have a statistically significant impact. The study found statistical improvements in several symptomatic outcomes, inflammatory markers (IL-6, CRP, NLR), and radiological changes (HRCT scores). 

Ayurveda's principles, like dosha (pathophysiological attributes) imbalances, agni (gut and cellular metabolism) disruption, and exogenous disease causes, can be applied to COVID-19. The disease primarily presents as *Sannipāta jvara* (complicated fever) with respiratory involvement and secondary complications. Gastrointestinal and immune factors also contribute. Disease progression shifts from *Vata-Kapha *predominant *Sannipāta jvara* to *Vata-Pitta* predominant *Sannipāta jvara* (pathological phenotypes) dominance, affecting vital organs (heart, lungs, kidney, brain, etc.). Abnormal immune responses result from *Tridosha* (pathophysiological attributes), *Rakta* (blood and related tissues), and *Ojus* (adaptive and self-regulatory immune system response) afflictions. This host-centric approach offers potential for reducing COVID-19 mortality and improving clinical outcomes, potentially reshaping epidemic management [35].

Ten herbal components, including *Ocimum sanctum, Curcuma longa, Tinospora cordifolia, Piper nigrum, Zingiber officinale, Syzygium aromaticum, Elettaria cardamomum, Citrus limon,* and *Withania somnifera,* which have been a part of the polyherbal formulations administered as our study intervention, have been found to exhibit antiviral effects by regulating viral replication and proliferation within host cells in an in-vivo study [36]. Furthermore, in a molecular docking study by Shree et al. bioactive metabolites derived from three ayurvedic herbs namely, *Withania somnifera, Tinospora cordifolia, *and* Ocimum sanctum,* have shown promising potential in enhancing immune function against infections [37].

The incorporation of Ayurvedic treatment, including herbal remedies, alongside standard care in the early stages of COVID-19 infection demonstrated notable improvements in symptoms such as fever, diarrhea, loss of taste, cough, shortness of breath, and headache when compared to the standard care-only group. These results are consistent with our earlier study on COVID-19 patients who have Type II diabetes and experience mild disease severity [33]. Despite the limited number of patients available on day 60, it is noteworthy that the Ayurveda add-on group exhibited statistically significant enhancements in symptoms commonly associated with long-term COVID-19, including anosmia, loss of taste, shortness of breath, general weakness, and headache. These findings suggest that Ayurvedic therapy may offer clinical benefits in mitigating or preventing symptoms associated with long-term COVID-19, warranting further investigation.

Biomarkers, such as the Neutrophil Lymphocyte Ratio (NLR), are reliable indicators of disease severity in COVID-19 patients [38,39]. Studies showed that excessive activation of neutrophils and their migration to the lungs can lead to lung injury and acute respiratory distress syndrome (ARDS) [40]. Our trial revealed significant NLR improvement in patients treated with Ayurveda plus standard of care (SOC) compared to SOC alone at 14 days, with a numerical trend continuing through 28 days. This was not seen at 60 days perhaps there were too few patients.

Eleven Ayurvedic herbs, namely *Phyllanthus emblica, Withania somnifera, Phyllanthus niruri, Vitis vinifera, Tinospora cordifolia, Curcuma longa, Andrographis paniculata, Solanum xanthocarpum, Allium sativum, Zingiber officinale*, and* Ocimum tenuiflorum*, used during the study, are known to regulate neutrophil function, foster immune balance and reduce thrombosis and coagulation by controlling neutrophil extracellular trap (NET) formation [41]. 

IL-6 has pro-inflammatory properties in both innate and adaptive immunity, and its levels can correlate with the degree of disease severity [42]. Through increasing oxidative stress, IL-6 can damage proteins, lipids, and DNA and impair the body’s structure and function, and Lim et al. propose that this effect might lead to the rapid progression of COVID-19 in patients with diabetes mellitus [43]. Our study yielded a statistically significant reduction in IL-6 levels in the add-on group when compared to the group receiving standard of care (SOC) alone at 14 days. This observation holds scientific significance as it can contribute to a more effective moderation of cytokine accumulation during the critical initial two weeks post-infection [44]. A recent study shows that nano-curcumin, as an anti-inflammatory herbal-based agent, modulates the increase of inflammatory cytokines, especially serum IL-1β and IL-6 mRNA expression and cytokine secretion in COVID-19 patients, which may reflect an improvement in clinical manifestation and overall recovery [45].

A comparative study revealed that symptomatic COVID-19-positive T2DM patients had significantly higher CRP and absolute neutrophil counts and lower counts of lymphocytes and eosinophils [46]. Another study showed that, compared with conventional treatment, the expression of CRP for patients with severe COVID-19 was significantly inhibited by an herbal Chinese decoction [47]. Ten studies reported the effect of herbal interventions on CRP levels with a net significant decrease after treatment [48]. In our study, the group that received add-on Ayurveda treatment during days 1-14 showed statistically significant improvements in CRP compared to SOC.

It is typically observed that individuals with pre-existing Type 2 diabetes mellitus (T2DM) who contact with COVID-19 experience a reduction in monocyte levels and alterations in their morphology [49-51]. In contrast, Ayurvedic medication in our study administered to patients during the inflammatory phase (days 0-14) and the chronic phase (days 14-28, 28-60) of COVID-19 appeared to result in different patterns. Notably, there was a significant increase in absolute monocyte counts during days 0-14 in the add-on Ayurveda group, probably indicating improved monocyte-macrophage-mediated opsonization compared to the control group [52,53]. The study employed formulations containing *Stereospermum suaveolens*. (Patala) root extract, which increased monocyte populations [54], and *Phyllanthus emblica*. (Aamlaki), known to stimulate the reticulo-endothelial system, activate polymorphonuclear and monocyte-macrophage systems, and enhance cell-mediated immunity in in-vivo studies [55].

According to a study by Rajamanickam et al., the progression of COVID-19 is correlated with an increase in the absolute counts of total monocytes and the frequencies of intermediate and non-classical monocytes from days 15-30 to days 61-90, leveling off thereafter [56]. In our study, we observed a significant reduction in monocytes at days 14 and 28 in the Ayurvedic add-on group. Modeling of the predicted trend curve suggests that the polyherbal combination of Ayurvedic medicines effectively managed the post-acute sequelae of COVID-19. However, further blinded, controlled studies are needed to validate these findings.

Contrary to common findings, a study by Junqueira et al. suggests that antibody-mediated uptake of SARS-CoV-2 by monocytes and macrophages triggers inflammatory cell death, leading to systemic inflammation that contributes to COVID-19 pathogenesis [57]. Despite an overall increase in the number of monocytes, the inflammatory markers IL-6 and CRP decreased significantly in our study group. This finding suggests that further investigation into the subsets of monocytes may provide insights into their impact on inflammatory markers and the pathogenesis of COVID-19.

COVID-19 can affect platelet function, increasing clotting risk [58]. In our study, the Ayurvedic add-on group showed stable platelet counts, while the standard care group exhibited an increase, albeit only at 60 days. Platelet count's clinical significance in long-term COVID-19 is unclear, with some studies suggesting lower platelet counts compared to healthy individuals [59,60] while others report higher counts [61-63], requiring further research for comprehension [64].

Elevated troponin levels are frequent in patients with COVID-19 and are significantly associated with fatal outcomes [9,65]. In a recent study involving COVID-19 patients with acute cardiac injury, 55.1% of patients at follow-up had persistently elevated troponin levels for a median of 2.5 months, thus indicating persistent cardiac damage even after the primary infection had resolved [66]. In our study, we found that the group receiving add-on ayurveda intervention showed a significant decrease in high sensitivity troponin values when compared to the standard of care at days 28 and 60, and this may denote a positive effect of add-on ayurveda.

In this study, we examined the use of CO-RADS severity scoring to assess the post-COVID syndrome in recovered patients with lower scores indicating improvement in lung HRCT [67]. After reviewing prior research, we observed that CO-RADS scores improved over time in COVID-19 patients who received antiviral treatment, while they remained stable or worsened in those who did not receive antiviral treatment [68-70]. In our study, despite the small sample size at 60 days, we noted a significant difference in CORADS scores between groups. Lung damage resolved faster in the Ayurveda add-on group compared to standard of care (SOC), suggesting potential benefits in reducing lung inflammation and improving the resolution of ground glass opacities. These findings are promising and merit further investigation.

Both groups displayed comparable trends in random blood sugar levels over the 60-day period as both received standard-of-care (SOC) interventions to manage potential effects on glycemic control.

While the acute COVID-19 pandemic is over, it can be anticipated that new strains of COVID-19 waves will occur, making this study useful for the future. Further, while COVID-19 is a particular virus, the concept of studying ayurvedic medications in well-controlled trials is worth considering. This study exhibits several notable strengths. It is one of the first controlled studies comparing Ayurveda add-on therapy to standard of care in oxygen-dependent, hospitalized, non-vaccinated, diabetic patients with moderate to severe COVID-19 with a 28-day follow-up plus some 60-day observations. The study addresses the safety and compatibility profiles of each intervention. The study design, characterized as an outcome-driven investigation, aligns with the epistemological principles of personalized medicine, particularly well-suited for elucidating the holistic therapeutic approach in Ayurveda. Additionally, the evaluation of a spectrum of innate immunological inflammatory biomarkers and 60-day radiological outcomes is of interest to understanding probable mechanisms of early resolution of lung lesions in the Ayurveda group.

The study also has limitations. While it was controlled, it lacked blinding and randomization as patients volunteered for their groups, introducing selection bias. Additionally, the open nature of the study may have led to evaluation bias. Difficulty in recruiting the planned number of participants was encountered due to challenges in identifying moderate to severe COVID-19 non-vaccinated patients with type 2 diabetes and NLR greater than four as the pandemic waned. Moreover, a high rate of loss to follow-up at 60 days was observed, attributed to concerns about being identified as COVID-19 patients in their communities, personal health issues, family obligations, lack of awareness regarding follow-up, spontaneous recovery during the study period, and reluctance to travel to the center. Consequently, the limited number of 60-day follow-ups impacted the confidence in the analyses at this time point. Fortunately, the 28-day follow-up yielded relatively robust results, and the limited 60-day analyses supported the findings at 28 days. Nevertheless, the presence of a larger effect size allows for the possibility of conducting a more extensive, well-controlled, double-blind study, whose findings could be generalized to a broader population.

## Conclusions

This exploratory, controlled, open-label study focused on non-vaccinated COVID-19 patients with pre-existing type II diabetes mellitus. The findings revealed statistically significant improvements in symptomatic outcomes and inflammation markers within a relatively short timeframe of 14 days, as well as sustained benefits over a 28-day period. Additionally, the small subset of subjects followed up at 60 days further supported the initial positive results, with encouraging trends observed in lung health assessments. Other hematological and coagulopathy parameters also showed significantly better responses in the add-on group. Importantly, minimal clinical AEs and no laboratory AEs were observed with the addition of Ayurveda. These promising findings underscore the necessity for a well-controlled, double-blind, randomized study in the future.

## Figures and Tables

**Figure 4:**
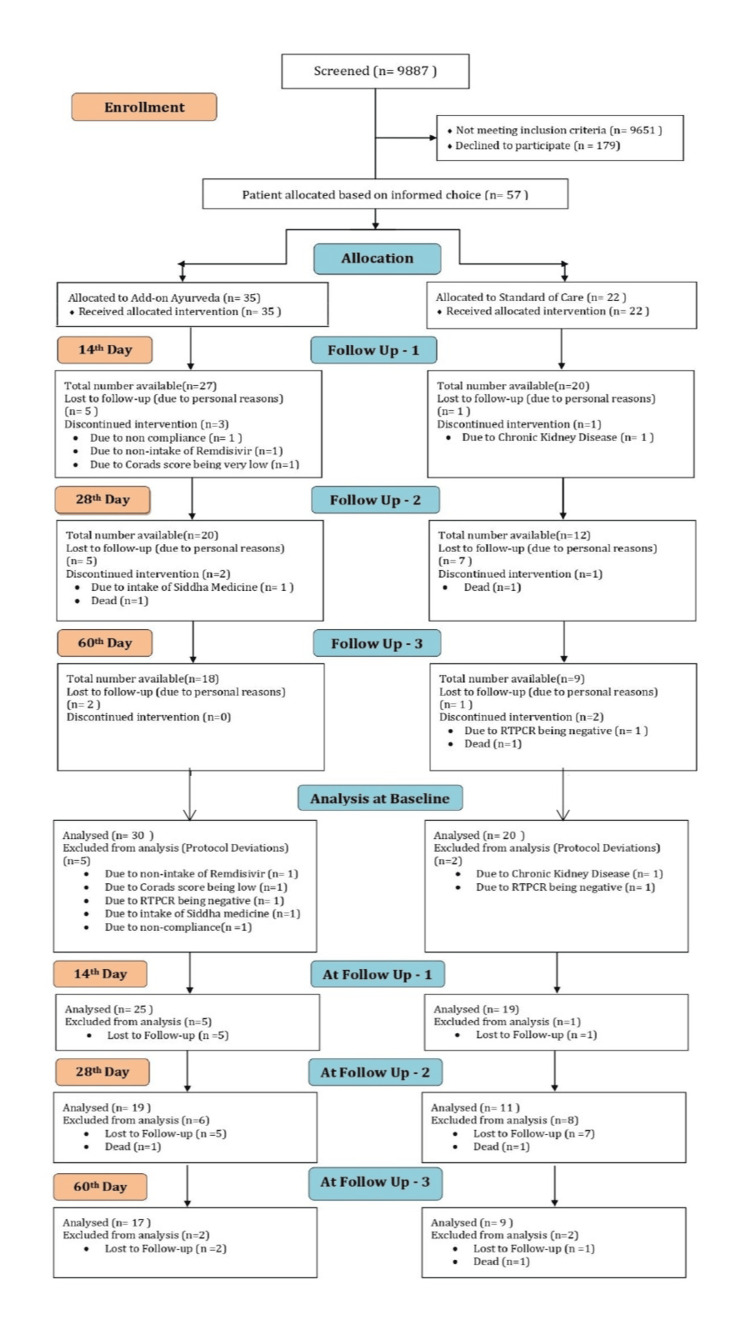
Flowchart as per TREND guidelines TREND Guidelines as given in the article: Haynes, A. B., Haukoos, J. S., & Dimick, J. B. (2021). TREND Reporting Guidelines for Nonrandomized/Quasi-Experimental Study Designs. JAMA surgery, 156(9), 879–880. https://doi.org/10.1001/jamasurg.2021.0552

**Figure 5:**
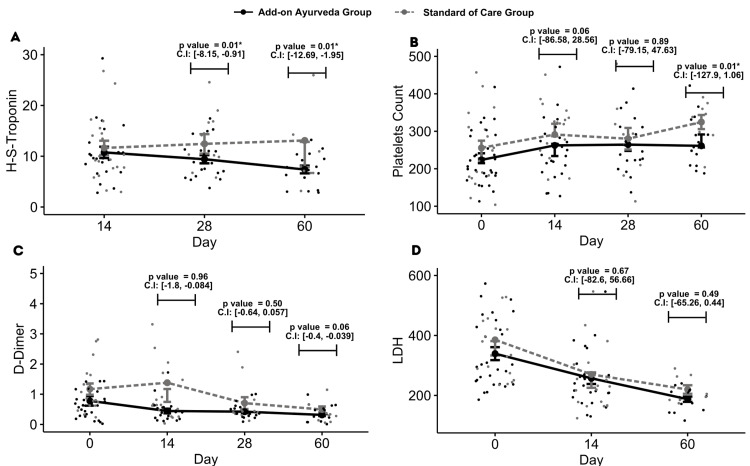
Changes in secondary outcomes A, B, C, D Depicts mean value of patients over time in the add-on ayurveda group and standard of care for H-S troponin, platelets count, D-dimer and LDH. *p-value computed using robust regression controlling for baseline differences between groups on the particular visit.

**Figure 6:**
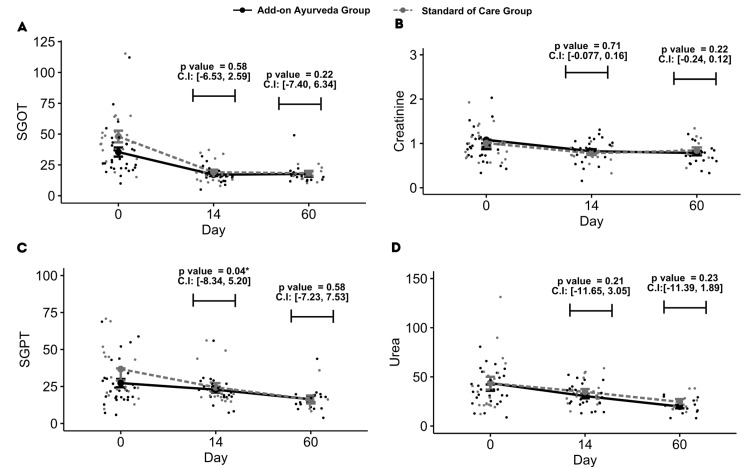
Changes in secondary outcomes: Safety markers A, B, C, D depicts mean value of patients over time in add-on ayurveda group and standard of care for SGOT, creatinine, SGPT and urea. *p-value computed using robust regression controlling for baseline differences between group on the particular visit.

## Appendices

**Table 6:** Sensitivity analyses: Treatment outcomes: Percentage of symptom presence over time (acute and chronic phase) (N = 36)a Gp 1: Add on Ayurveda Group, Gp 2: Standard of care group *Values at 95% Confidence Interval where the level of significance, α= 0.05. ^a ^Population with last observation carried forward for 20% of missing values lost to follow-up. ^b ^p-values assessed by Chi-square/Fisher’s test over 14 days. ^c ^p-values assessed using mixed effects logistic regression with treatment group and time point as covariates, while adjusting for baseline differences with the improvement in response as the outcome.

Parameters	Baseline		Day 14			Day 28			Day 60		p value between groups over time^c^
	Gp 1 (N = 22)	Gp 2 (N = 14)	Gp 1 (N = 22)	Gp 2 (N = 14)	p value between groups over 14 days^b^	Gp 1 (N = 22)	Gp 2 (N = 14)	p value between groups over 28 days^c^	Gp 1 (N = 22)	Gp 2 (N = 14)	
Anosmia	59.10%	35.70%	18.20%	14.30%	0.029*	13.60%	7.10%	0.036*	9.10%	7.10%	0.045*
Tastelessness	36.40%	35.70%	18.20%	17.10%	0.609	13.60%	0.00%	0.048*	4.50%	0.00%	0.427
Cough	78.60%	81.80%	28.60%	35.90%	0.633	14.30%	22.70%	0.648	14.30%	22.70%	0.176
Shortness of breath	54.50%	50.00%	22.70%	17.10%	0.676	18.20%	17.10%	0.785	9.10%	17.10%	0.045*
General Weakness	77.30%	71.40%	63.60%	50.00%	0.809	36.40%	35.70%	0.899	31.80%	42.90%	0.859
Headache	27.30%	21.40%	9.10%	0.00%	0.693	27.30%	0.00%	0.788	9.10%	7.10%	0.998
Confusion	9.10%	7.10%	0.00%	0.00%	-	0.00%	0.00%	-	4.50%	0.00%	-

**Table 7:** Sensitive analyses: Treatment outcomes: Percentage of symptom presence over time (acute phase) (N = 43)a Gp 1: Add on Ayurveda Group, Gp 2: Standard of Care Group *Values at 95% Confidence Interval where the level of significance, α= 0.05. ^a^ Population of cases completing Day 14. ^b^ p-values assessed by Chi-square/Fisher’s test over 14 days.

Parameters	Baseline		Day 14		p-value between groups over 14 daysᵃ
	Gp 1 (N = 24)	Gp 2 (N = 19)	Gp 1 (N = 24)	Gp 2 (N = 19)	
Fever	20.83%	10.53%	0.00%	0.00%	0.04*
Sore throat	8.33%	21.06%	20.83%	0.00%	0.06
Running nose	8.33%	0.00%	0.00%	5.26%	-
Irritability	16.67%	21.06%	4.16%	5.26%	0.99
Nausea	20.83%	31.58%	4.16%	0.00%	0.99
Diarrhoea	8.33%	42.08%	4.16%	0.00%	0.02*

**Table 8:** Sensitivity analyses: Treatment outcomes over time, by group (N = 36)a Gp 1: Add on Ayurveda Group, Gp 2: Standard of Care Group ^a^ Population with last observation carried forward for 20% of missing values lost to follow-up. * Values at a 95% confidence interval where the level of significance, α= 0.05. ^b^ p-values assessed using the method of robust regression controlled for baseline differences with treatment group as covariate has been used to test differences between the treatment groups unless otherwise noted. ^c^ p-values assessed using ANCOVA at 95 CI. ** Parameter not assessed as per the protocol; NA: Not Applicable

Parameter	Days of therapy	Gp 1			Gp 2			p-value	Estimate
		N	Mean	SD	N	Mean	SD		
Interleukin-6^b^	Baseline	22	65.9	43.4	14	56.3	43.6	-	-
	14	22	6.3	6.8	14	18.2	29.6	0.029*	-14.58
	28	NA**	NA**	NA**	NA**	NA**	NA**	NA**	NA**
	60	22	5.3	5.6	14	5.9	8.5	0.58	0.14
C-reactive protein^b^	Baseline	22	7.5	5.7	14	8.9	7.0	-	-
	14	22	2.4	4.7	14	6.5	8.9	0.037*	-3.65
	28	NA**	NA**	NA**	NA**	NA**	NA**	NA**	NA**
	60	22	1.0	1.7	14	1.9	4.4	0.67	0.78
Neutrophils-Lymphocytes Ratio^b^	Baseline	22	7.8	3.9	14	7.9	3.5	-	-
	14	22	6.6	3.7	14	8.5	4.4	0.04*	-1.74
	28	22	3.9	2.3	14	5.1	3.9	0.17	-1.16
	60	22	3.4	2.3	14	4.8	3.1	0.11	-1.26
HS- Troponin^b^	Baseline	NA**	NA**	NA**	NA**	NA**	NA**	NA**	NA**
	14	22	10.6	5.6	14	11.7	7.2	-	-
	28	22	9.1	3.9	14	13.7	12.7	0.01*	-3.5
	60	22	7.7	3.8	14	11.8	8.7	0.03*	-3.4
HRCT CORADS score^c^	Baseline	16	13.4	4.6	9	13.8	2.8	-	-
	14	NA**	NA**	NA**	NA**	NA**	NA**	NA**	NA**
	28	NA**	NA**	NA**	NA**	NA**	NA**	NA**	NA**
	60	16	6.3	5.0	9	10.9	5.9	0.046*	-4.8

**Table 9:** Details of adverse events

Clinical findings of Adverse Events	Total Number of patients with adverse events							
	Gp 1 (n = 4)				Gp 2 (n = 3)			
	Day	Freq	Grade	Related to	Day	Freq	Grade	Related to
Burning sensation in eyes (ENT)	29	1	Mild	Disease				
Pedal edema (CV)	27	1	Mild	Drug	16	1	Moderate	Drug
Blister formation on lips (GI)	19	1	Mild	Disease				
Breathing Difficulty (RES)	19	1	Mild	Disease				
Constipation (GI)	19	1	Mild	Disease				
Sweating (GEN)	19	1	Moderate	Disease				
Frequent Urination (UT)	19	1	Mild	Disease				
Itching (SKIN)	19	1	Mild	Disease				
Traces of blood in phlegm (RES)	1	1	Mild	Disease				
Serious Adverse Events								
Tachypnoea (RES)	5	1	Severe	Disease				
Death	15	1	Severe	Disease	18,26	2	Severe	Disease

**Table 10:** Secondary outcomes: chronic phase markers * Values at a 95% confidence interval where the level of significance, α= 0.05 The method of robust regression controlled for baseline differences with treatment group as a covariate has been used to test differences between the treatment groups. ** Parameter not assessed on day 28 as per the protocol; NA: Not Applicable

Parameter	Days of therapy	Gp 1			Gp 2			p-value	Estimate
		N	Mean	SD	N	Mean	SD		
Computed Tomography score	0	29	12.66	4.38	19	12.21	4.45	-	-
	60	16	6.75	5.43	9	10.89	5.9	0.04*	-4.80
HS Troponin	0	12	23	29.2	7	18.44	17.24	-	-
	14	24	10.76	5.45	20	11.63	6.34	0.71	-0.97
	28	21	9.41	3.7	12	13.94	6.56	0.01*	-6.59
	60	16	7.14	3.06	9	14.46	9.69	0.35	-3.36
Lactate dehydrogenase	0	28	339.29	114.49	19	385.47	126.05	-	-
	14	25	257.03	94.53	19	270	134.44	0.67	11.88
	28	NA**	NA**	NA**	NA**	NA**	NA**	NA**	NA**
	60	16	187.37	35.48	9	219.78	42.6	0.49	-15.29
D Dimer	0	30	0.78	0.87	19	1.17	0.81		
	14	24	0.44	0.37	19	1.38	2.04	0.96	-0.03
	28	20	0.42	0.23	13	0.71	0.71	0.5	0.04
	60	16	0.31	0.15	9	0.53	0.29	0.06	-0.11
Platelets	0	30	223.63	73.84	20	255.85	116.94		
	14	25	262.36	98.82	19	291.37	86.47	0.06	-48.29
	28	19	264.42	72.96	11	280.18	95.4	0.89	-4.65
	60	16	261.25	83.03	9	324.67	56.25	0.01*	-64.28
Absolute Monocytes	0	30	0.5	0.48	20	0.51	0.31	-	-
	14	25	0.94	0.45	19	0.66	0.33	0.03*	0.245
	28	19	0.62	0.19	11	0.45	0.18	0.01*	0.182
	60	16	0.61	0.25	9	0.47	0.15	0.11	0.13
Percentage Monocytes	0	30	6.18	4.23	20	7.43	2.81		
	14	25	6.59	2.6	19	7.11	4.27	0.72	0.28
	28	19	7.94	1.96	11	6.97	1.73	0.01*	1.38
	60	16	7.55	2.14	9	6.37	0.9	0.20	0.73
Absolute Lymphocytes	0	30	0.9	0.31	20	0.85	0.28	-	-
	14	25	1.5	0.65	19	1.68	1.18	0.54	-0.12
	28	19	1.65	0.51	11	1.77	0.73	0.94	-0.02
	60	16	1.92	0.60	9	1.93	0.52	0.70	-0.08
Percentage Lymphocytes	0	30	12.03	3.66	20	12.48	3.78	-	-
	14	25	14.45	7.96	19	14.92	8.65	0.58	-1.626
	28	19	20.91	5.2	11	24.37	6.25	0.18	-3.05
	60	16	24.30	5.79	9	24.59	6.32	0.61	-1.32
Absolute Neutrophils	0	30	6.61	2.89	20	5.83	2.23	-	-
	14	25	8.99	3.18	19	10.01	4.59	0.62	-0.03
	28	19	5.33	1.44	11	4.67	0.89	0.53	0.03
	60	16	5.14	1.29	9	5.4	1.49	0.92	-0.06
Percentage Neutrophils	0	30	81.33	6.07	20	80.19	5.42	-	-
	14	25	77.37	9.72	19	77.06	10	0.88	-0.003
	28	19	67.06	5.56	11	65.45	5.78	0.11	0.0248
	60	16	64.53	6.47	9	64.87	8.3	0.92	-0.17
Ferritin	0	30	589.4	477.3	20	805.7	523	-	
	14	25	369.9	364.2	19	456.8	341.8	0.38	-39.36
	28	NA**	NA**	NA**	NA**	NA**	NA**	NA**	NA**
	60	16	167.23	83.03	9	172.9	151.6	0.44	32.31

**Table 11:** Hematological parameters * Values at a 95% confidence interval where the level of significance, α= 0.05 The method of robust regression controlled for baseline differences with treatment group as a covariate has been used to test differences between the treatment groups.

Parameter	Days of Therapy	Gp 1			Gp 2			p-value	Estimate
		N	Mean	SD	N	Mean	SD		
White Blood Cells	0	30	8.03	3.16	20	7.37	2.74	-	-
	14	25	11.36	3.29	19	12.61	4.68	0.83	-0.22
	28	19	7.93	1.82	11	7.13	1.45	0.34	0.61
	60	16	7.94	1.80	9	8.21	1.93	0.98	-0.01
Red Blood Cells	0	30	4.51	0.62	20	4.26	0.6	-	-
	14	25	4.69	0.54	19	4.31	0.73	0.05*	0.21
	28	19	4.47	0.36	11	4.04	0.68	0.24	0.16
	60	16	4.54	0.41	9	4.37	0.62	0.50	0.09
Random Blood Sugar	0	25	259.64	107	18	254.83	124.58	-	-
	14	24	311.17	91.16	18	355.37	152.19	0.18	-56.03
	28	18	242.61	76.88	12	254.08	91.42	0.17	-43.47
	60	16	225.12	64.57	9	244.22	125.11	0.62	-20.83
Hemoglobin	0	30	12.82	1.66	20	12.3	1.84	-	-
	14	25	13.26	1.77	19	12.34	1.8	0.09	0.52
	28	19	12.86	1.23	11	11.87	2.22	0.4	0.35
	60	16	13.12	1.05	9	12.88	1.95	0.57	0.20

**Table 12:** Liver and renal markers * Values at a 95% confidence interval where the level of significance, α= 0.05 The method of robust regression controlled for baseline differences with treatment group as a covariate has been used to testing differences between the treatment groups. ** Parameter not assessed on Day 28 as per the protocol; NA – Not Applicable

Parameter	Days of therapy	Gp 1			Gp 2			p-value	Estimate
		N	Mean	SD	N	Mean	SD		
Aspartate transaminase	0	30	35.42	20.2	20	48.05	21.17	-	-
	14	24	17.09	5.7	18	19.06	8.9	0.58	1.12
	28	NA**	NA**	NA**	NA**	NA**	NA**	NA**	NA**
	60	16	17.69	8.84	9	18.22	6.02	0.22	-2.46
Alanine transaminase	0	30	27.27	15.3	20	36.75	23.65	-	-
	14	24	22.85	9.5	18	24.42	12.21	0.04*	-2.54
	28	NA**	NA**	NA**	NA**	NA**	NA**	NA**	NA**
	60	16	16.37	8.78	9	16.22	8.14	0.58	1.28
Alkaline phosphatase	0	29	69.21	28.1	20	91.15	30.15	-	-
	14	24	84.53	21.5	18	104.51	23.89	0.07	-11.51
	28	NA**	NA**	NA**	NA**	NA**	NA**	NA**	NA**
	60	16	83.06	26.60	9	84.44	26.02	0.94	-0.75
Direct Bilirubin	0	30	0.21	0.1	20	0.23	0.11	-	-
	14	24	0.22	0.1	18	0.21	0.1	0.79	0.01
	28	NA**	NA**	NA**	NA**	NA**	NA**	NA**	NA**
	60	16	0.18	0.06	9	0.19	0.14	0.09	0.04
Total Bilirubin	0	30	0.52	0.2	20	0.49	0.18	-	-
	14	24	0.53	0.3	18	0.52	0.18	0.52	-0.04
	28	NA**	NA**	NA**	NA**	NA**	NA**	NA**	NA**
	60	16	0.42	0.18	9	0.47	0.31	0.24	0.07
Urea	0	30	43.62	30.1	20	43.6	28.98	-	-
	14	25	30.48	11.5	18	34.78	12.15	0.21	-3.87
	28	NA**	NA**	NA**	NA**	NA**	NA**	NA**	NA**
	60	16	19.81	7.11	9	24.56	8.71	0.23	-4.24
Albumin	0	29	3.46	0.4	20	3.45	0.44	-	-
	14	24	3.88	0.3	18	3.61	0.44	0.02*	0.28
	28	NA**	NA**	NA**	NA**	NA**	NA**	NA**	NA**
	60	16	4.33	0.23	9	4.34	0.27	0.76	-0.03
Total Protein	0	29	6.9	0.6	20	7.19	0.6	-	-
	14	24	6.65	0.5	18	6.73	0.7	0.84	-0.04
	28	NA**	NA**	NA**	NA**	NA**	NA**	NA**	NA**
	60	16	6.86	0.40	9	7.28	0.32	0.01*	-0.46
Creatinine	0	30	1.08	0.9	20	1.01	0.37	-	-
	14	25	0.82	0.2	18	0.78	0.17	0.71	0.02
	28	NA**	NA**	NA**	NA**	NA**	NA**	NA**	NA**
	60	16	0.78	0.21	9	0.84	0.2	0.22	-0.08

**Table 13:** Vitals * Values at a 95% confidence interval where the level of significance, α= 0.05 The method of robust regression controlled for baseline differences with treatment group as a covariate has been used to test differences between the treatment groups.

Parameter	Day of therapy	Gp 1				Gp 2				p-value	Estimate
		N	Q1	Median	Q3	N	Q1	Median	Q3		
Temperature	0	29	36.1	36.5	36.9	18	35.575	36.3	36.625	-	-
	14	23	35.8	36.5	36.6	15	35.6	36	36.5	0.06	0.44
	28	21	36.2	36.4	36.6	10	35.2	36.25	36.45	0.84	0.03
	60	17	34.3	36	36.45	9	36.035	36.3	36.6	0.90	0.02
Pulse rate	0	30	74.7	86.5	95.25	17	75.5	85	97	-	-
	14	23	85	94	102	16	83	101.5	123.75	0.02*	-11.77
	28	21	84	97	104	10	88	94	102.75	0.84	-0.87
	60	17	86	91	96	9	72	90	97	0.81	-0.85
Respiratory rate	0	25	22.5	25	29	18	20	22	26.75	-	-
	14	22	19.5	22	25.25	13	22	24	25	0.25	-1.55
	28	16	18	22.5	26	8	20.5	22	24	0.45	-1.44
	60	15	22	24	27	8	22.5	24	25.5	0.99	-0.02
Systolic BP	0	29	118	128	140	19	130	135	147	-	-
	14	22	109.	125.5	136.7	15	120	128	149	0.84	-1.41
	28	21	119.5	127	142	11	109	132	150	0.65	3.59
	60	17	126.5	132	137	9	114	128	130.5	0.04*	9.27
Diastolic BP	0	29	69	80	84.5	19	70	81	90	-	-
	14	22	72.75	81	84.2	15	75	83	91	0.18	-5.35
	28	21	74	80	89	11	70	77	91	0.58	2.41
	60	17	74	80	88.5	9	73	79	84	0.19	-4.94
Oxygen Saturation	0	28	89	94	96.7	16	92.2	94.5	96.7	-	-
	14	23	94	97	98	16	95	96	96.7	0.18	1.06
	28	21	95	96	97	11	95	96	98	0.61	-0.35
	60	17	95	96	97	9	96	97	99	0.63	2.03

**Table 14:** Survival analysis p-value computed using the Log-rank test at a 95% confidence interval where the level of significance, α = 0.05

	Gp 1	Gp 2
N=50	30	20
Number of ICU Admissions	2	2
Number of deaths	1	2
Rate of Survival (in %)	96.7	90
Rate of ICU Admissions (in %)	6.67	10
Death Rate (in %)	3.33	10
P value	0.38	

**Table 15:** Rationality behind the formulation used in COVID-19 during Baseline-14 days (Ama or Pachyamana Jwara phase) in add-on Ayurveda Group (Group 1) – Ayurvedic Medicine Track *n represents the count of patients who received the specified medication within the given timeframe +Dosage and frequency were decided based on the Ayurveda physician’s clinical judgement

Medicine Name	Rationale	Dosage^+^	n (%)*	Frequency of intake^+^
Kashyam				
Bharangyadi Kashayam	Sannipataja jwara(complicated fevers involving all three doshas), vishamajwara(intermittent fever), jeerna jwara(chronic fever), shirograha(headache), parshwashoola (pain in the flanks of the chest), hrith shola(chest pain around the heart), kasa(cough), shwasa(dyspnea) , tandra(semi-comatose condition) and agnimandhya(low metabolism).	7.5 - 15 ml	21 (100%)	Once/Twice
Indukantham Kashayam	Vata predominant type of fever associated with cough, breathing difficulty and fluid accumulation.	7.5 - 15 ml	20 (95.2%)	Once/Twice
Panchathikthakam Kashayam	All eight type of fever as classified in Ayurveda associated with Kasa swasa.	7.5 ml	15 (71.4%)	Once/Twice
Nayopayam Kashayam	Given during acute phase of COVID-19 with acute dyspnea, bloatedness, reverse peristalsis, belching associated with chest discomfort.	7.5 ml	12 (57.1%)	Once/Twice
Drakshadi Kashyam	In Chronic fever and Vomiting	7.5 - 15 ml	8 (38.1%)	Once/Twice
Guduchyadi Kashayam	In fever, vomiting, renal disorders, migraine, and diabetes.	7.5 - 20 ml	7 (33.3%)	Once/Twice
Gulika/Pills				
Gorochanadi Gulika	Fever associated with giddiness, nausea, breathlessness, General weakness, headache, Asthma, hiccups, cough, Hemiplegia and Ansomia.	100 mg	21 (100%)	Once/Twice/four
Suvarnamukatadi	Fever, vertigo, fatigue, loss of smell, and semi-comatose conditions.	27 - 54 mg	17 (81%)	Once/Twice/four
Swasanandam	Acute dyspnea.	50 - 200 mg	16 (76.2%)	Once/Twice
Vilwadi Gulika	In Vata Kapha fever associated with indigestion, loose motion, vomiting and symptoms of toxicity.	325 - 650 mg	3 (14.3%)	Once/Twice
Bhasmam				
Pravala Bhasmam	Acute dyspnea associated with hyperacidity and to iniate innate immune response.	125 - 250 mg	16 (76.2%)	Every 3 hours/Twice/once
Lehyam				
Agasthyarasayanam	Given both in acute and chronic condition of Cough, Dyspnoea, Rhinitis, Hiccup, Fever, Lack of appetite in COVID patients.	5 - 6 g	16 (76.2%)	Every 3 hours/Twice/once
Vidaryadi Lehyam	Given during both acute and chronic COVID-19 patients showing symptoms of fatigue, weight loss, myalgia, shortness of breath having moderate or good digestive activity.	5 - 6 g	3 (14.3%)	Every 3 hours/Twice
Brahmarasayana	Given both in acute and chronic condition of Cough, Dyspnoea, Rhinitis, Hiccup, Fever, Lack of appetite in COVID patients.	5 - 6 g	1 (4.8%)	Every 3 hours/Twice/once
Gritham				
Baladi Gritham	Given during both acute and chronic COVID-19 patients showing symptoms of fatigue, shortness of breath, persistent reoccurring fever, and lung consolidation.	1/2 - 5 - 6 g	3 (14.3%)	Once/Twice

**Table 16:** Rationality behind the used formulation in COVID-19 during Baseline-14 days (Ama or Pachyamana Jwara phase) in Standard of care Group (Group 2) – Allopathic Medicine Track *n represents the count of patients who received the specified medication within the given timeframe +Dosage and frequency were decided based on the Allopathic physician’s clinical judgement

Medicine Name	Rationale	Dosage+	n (%)*	Frequency of intake+
Enoxaparin	An anticoagulant used to prevent harmful blood clots	0-4-0.6 cc	9 (100%)	Once-Twice
Remdesivir	This is a broad-spectrum antiviral medicine. It is used for the treatment of suspected or laboratory-confirmed coronavirus disease 2019 (COVID-19) in adults and children hospitalized with severe disease.	200 mg via infusion loading dose followed by 100 mg per day for 5-10 days	9 (100%)	Once
Metformin	Anti-diabetic drugs	500mg	7 (77.7%)	Once-Twice
Zac D	Vitamin supplements	-	7 (77.8%)	Once-Twice
Methylprednisolone	Steroids. It is used to treat different conditions including rheumatic disorder, asthma, severe allergic reactions and conditions that affect your eyes and skin.	1-2mg /kg /day <=7 days	5 (55.6%)	Once-Twice
Pantoprazole	Antacids	40mg	5 (55.6%)	Once
Chlorpheniramine Maleate	Cough remedial Medication	10-15 ml	4 (44.4%)	Once-Twice
Pantoprazole	Antacids	40mg	4 (44.4%)	Once-Twice
Paracetamol	Helps relieve pain and fever by blocking the release of certain chemical messengers responsible for fever and pain.	650mg	3 (33.3%)	Once
Paracetamol	Helps relieve pain and fever by blocking the release of certain chemical messengers responsible for fever and pain.	650mg	3 (33.3%)	Thrice
Ondansetron	Used to control nausea and vomiting	4-11mg	2 (22.2%)	Once
Methylprednisolone	Steroids. It is used to treat different conditions including rheumatic disorder, asthma, severe allergic reactions and conditions that affect your eyes and skin.	1-2mg /kg /day <=7 days	2 (22.2%)	Twice
Cefoperazone and Sulbactam	It is prescribed to treat various types of bacterial infections. It fights against the microorganisms to prevent their growth and further spread of the infection.	1.5 -3 g/dose	2 (22.2%)	Once-Twice
Chlorpheniramine Maleate	Cough remedial Medication	10-15 ml	2 (22.2%)	Twice
Glimepiride	Anti-diabetic drugs	1-2mg/dose	2 (22.2%)	Once-Twice
Methylprednisolone	Steroids. It is used to treat different conditions including rheumatic disorder, asthma, severe allergic reactions and conditions that affect your eyes and skin.	1-2mg /kg /day <=7 days	2 (22.2%)	Once
Ursodeoxycholic acid	Dissolves gallstones and prevents them from forming.	150-300mg	2 (22.2%)	Twice

**Table 17:** Rationality behind the used formulation in COVID-19 during 14 days-28 days (Jirna Jwara phase) in Add-on Ayurveda Group (Group 1) – Ayurvedic Medicine Track *n represents the count of patients who received the specified medication within the given timeframe +Dosage and frequency were decided based on the Ayurveda physician’s clinical judgement

Medicine Name	Rationale	Dosage^+^	n (%)*	Frequency of intake^+^
Kashyam				
Bharangyadi Kashayam	Sannipataja jwara(complicated fevers involving all three doshas), vishamajwara(intermittent fever), jeerna jwara(chronic fever), shirograha(headache), parshwashoola (pain in the flanks of the chest), hrith shola(chest pain around the heart), kasa(cough), shwasa(dyspnea) , tandra(semi-comatose condition) and agnimandhya(low metabolism).	7.5 - 15 ml	20 (95.2%)	Once/Twice
Indukantham Kashayam	Vata predominant type of fever associated with cough, breathing difficulty and fluid accumulation.	7.5 - 15 ml	18 (85.7%)	Once/Twice
Nayopayam Kashayam	Given during acute phase of COVID-19 with acute dyspnea, bloatedness, reverse peristalsis, belching associated with chest discomfort.	7.5 ml	11 (52.4%)	Once/Twice
Panchathikthakam Kashayam	All eight type of fever as classified in Ayurveda associated with Kasa swasa.	7.5 ml	6 (28.6%)	Once/Twice
Drakshadi Kashyam	In Chronic fever and Vomiting	7.5 - 15 ml	5 (23.8%)	Once/Twice
Guduchyadi Kashayam	In fever, vomiting, renal disorders, migraine, and diabetes.	7.5 ml	4 (19%)	Once/Twice
Rasonadi kashayam	Given in both acute and chronic COVID-19 cases where cardiovascular stress, and coagulopathy symptoms are high.	7.5 ml	2 (9.5%)	Twice
Gulika/Pills				
Gorochanadi Gulika	Fever associated with giddiness, nausea, breathlessness, General weakness, headache, Asthma, hiccups, cough, Hemiplegia and Ansomia.	100 mg	16 (76.2%)	Once/Twice/four
Suvarnamukatadi	Fever, vertigo, fatigue, loss of smell, and semi-comatose conditions.	27 - 54 mg	15 (71.4%)	Once/Twice/four
Swasanandam	Acute dyspnea.	50 - 200 mg	9 (42.9%)	Once/Twice
Bhasmam				
Pravala Bhasmam	Acute dyspnea associated with hyperacidity and to iniate innate immune response.	125 - 250 mg	16 (76.2%)	Every 3 hours/Twice/once
Lehyam				
Agasthyarasayanam	Given both in acute and chronic condition of Cough, Dyspnoea, Rhinitis, Hiccup, Fever, Lack of appetite in COVID patients.	5 - 6 g	17 (81%)	Every 3 hours/Twice/once
Brahmarasayana	Given both in acute and chronic condition of Cough, Dyspnoea, Rhinitis, Hiccup, Fever, Lack of appetite in COVID patients.	5 - 6 g	6 (28.6%)	Every 3 hours/Twice/once
Vidaryadi Lehyam	Given during both acute and chronic COVID-19 patients showing symptoms of fatigue, weight loss, myalgia, shortness of breath having moderate or good digestive activity.	5 - 6 g	4 (19%)	Every 3 hours/Twice
Gritham				
Baladi Gritham	Given during both acute and chronic COVID-19 patients showing symptoms of fatigue, shortness of breath, persistent reoccurring fever, and lung consolidation.	1/2 - 5 - 6 g	14 (66.7%)	Once/Twice
Dhanwantharam Gritham	Given in COVID-19 patients with diabetes after acute inflammatory phase with persistent symptoms of shortness of breath, dyspnea, lung consolidation, and brain fog.	5 - 6 g	1 (4.8%)	Once

**Table 18:** Rationality behind the used formulation in COVID-19 during 14 days-28 days (Jirna Jwara phase) in Standard of care Group (Group 2) – Allopathic Medicine Track *n represents the count of patients who received the specified medication within the given timeframe +Dosage and frequency were decided based on the Allopathic physician’s clinical judgement

Medicine Name	Rationale	Dosage+	n (%)*	Frequency of intake+
Metformin	Anti-diabetic drugs	500mg	7 (77.7%)	Once-Twice
Apixaban	Antihypertensive drugs	2.5mg	2 (22.2%)	Twice
Paracetamol	Helps relieve pain and fever by blocking the release of certain chemical messengers responsible for fever and pain.	650mg	2 (22.2%)	SOS
Aspirin	An antiplatelet medicine used to treat and prevent heart attacks, strokes, and heart-related chest pain (angina).	75mg	2 (22.2%)	Once
Glimepiride	Anti-diabetic drugs	1-2mg/dose	2 (22.2%)	Once-Twice

**Table 19:** Rationality behind the used formulation in COVID-19 during 28 days-60 days (Punaravartaka Jwara phase) in Add-on Ayurveda Group (Group 1) – Ayurvedic Medicine Track *n represents the count of patients who received the specified medication within the given timeframe +Dosage and frequency were decided based on the Ayurveda physician’s clinical judgement

Medicine Name	Rationale	Dosage^+^	n (%)*	Frequency of intake^+^
Kashyam				
Bharangyadi Kashayam	Sannipataja jwara(complicated fevers involving all three doshas), vishamajwara(intermittent fever), jeerna jwara(chronic fever), shirograha(headache), parshwashoola (pain in the flanks of the chest), hrith shola(chest pain around the heart), kasa(cough), shwasa(dyspnea) , tandra(semi-comatose condition) and agnimandhya(low metabolism).	7.5 - 15 ml	19 (90.5%)	Once/Twice
Indukantham Kashayam	Vata predominant type of fever associated with cough, breathing difficulty and fluid accumulation.	7.5 - 15 ml	15 (71.4%)	Once/Twice
Nayopayam Kashayam	Given during acute phase of COVID-19 with acute dyspnea, bloatedness, reverse peristalsis, belching associated with chest discomfort.	7.5 ml	8 (38.1%)	Once/Twice
Drakshadi Kashyam	In Chronic fever and Vomiting	7.5 - 15 ml	6 (28.6%)	Once/Twice
Panchathikthakam Kashayam	All eight type of fever as classified in Ayurveda associated with Kasa swasa.	7.5 ml	3 (14.3%)	Once/Twice
Guduchyadi Kashayam	In fever, vomiting, renal disorders, migraine, and diabetes.	7.5 ml	2 (9.5%)	Once/Twice
Rasonadi kashayam	Given in both acute and chronic COVID-19 cases where cardiovascular stress, and coagulopathy symptoms are high.	7.5 ml	2 (9.5%)	Twice
Patolakadurohinyadi Kashayam	In Kapha Pitta type of fever associated with Vomiting, Loss of appetite, skin rashes and symptoms of toxicity.	7.5 ml	1 (4.8%)	Twice
Gulika/Pills				
Suvarnamukatadi	Fever, vertigo, fatigue, loss of smell, and semi-comatose conditions.	27 - 54 mg	14 (66.7%)	Once/Twice/four
Gorochanadi Gulika	Fever associated with giddiness, nausea, breathlessness, General weakness, headache, Asthma, hiccups, cough, Hemiplegia and Ansomia.	100 mg	12 (57.1%)	Once/Twice/four
Swasanandam	Acute dyspnea.	50mg	3 (14.3%)	Once/Twice
Bhasmam				
Pravala Bhasmam	Acute dyspnea associated with hyperacidity and to iniate innate immune response.	125 - 250 mg	12 (57.1%)	Every 3 hours/Twice/once
Lehyam				
Agasthyarasayanam	Given both in acute and chronic condition of Cough, Dyspnoea, Rhinitis, Hiccup, Fever, Lack of appetite in COVID patients.	5 - 6 g	12 (57.1%)	Every 3 hours/Twice/once
Vidaryadi Lehyam	Given during both acute and chronic COVID-19 patients showing symptoms of fatigue, weight loss, myalgia, shortness of breath having moderate or good digestive activity.	5 - 6 g	6 (28.6%)	Every 3 hours/Twice
Brahmarasayana	Given both in acute and chronic condition of Cough, Dyspnoea, Rhinitis, Hiccup, Fever, Lack of appetite in COVID patients.	5 - 6 g	4 (19%)	Every 3 hours/Twice/once
Gritham				
Baladi Gritham	Given during both acute and chronic COVID-19 patients showing symptoms of fatigue, shortness of breath, persistent reoccurring fever, and lung consolidation.	1/2 - 5 - 6 g	18 (85.7%)	Once/Twice
